# Menopause and Quality of Life Among Greek Women in Rural Areas

**DOI:** 10.7759/cureus.72716

**Published:** 2024-10-30

**Authors:** Giannoula A Kyrkou, Eleni Karozi, Anastasia Bothou, Anna Deltsidou, Athina Diamanti, Nikoleta Tsinisizeli, Aikaterini Lykeridou, Antigoni Sarantaki

**Affiliations:** 1 Midwifery, Faculty of Health and Care Sciences, University of West Attica, Athens, GRC; 2 Οbstetrics and Gynecology Outpatient Clinics, Aliveri State Health Center, Aliveri, GRC; 3 Neonatal Intensive Care Unit, General State Hospital of Nikaia “Agios Panteleimon”, Athens, GRC

**Keywords:** alternative therapies, menopausal symptoms, menopause, physical exercise, quality of life

## Abstract

Introduction

The World Health Organization defines menopause as the permanent termination of ovarian function and the absence of menstruation for a minimum of 12 months. A woman's quality of life and health may be affected by menopause, a physiological condition that is typically accompanied by vasomotor, physical, and psychological disorders. Also, menopausal women experience a wide range of psychological symptoms, such as irritability, anxiety, sadness, and depression. Additional factors such as her marital status, financial level, educational level, physical activity, temperament, and attitude toward life greatly influence the intensity and frequency of the menopausal symptoms she will experience, as well as the level of quality of her life.

Aim

The purpose of this study is to evaluate the symptoms of menopause and investigate their impact on women's quality of life.

Materials and methods

The research was carried out in the wider region of Southern Evia. One hundred fifty menopausal women participated, and the anonymous Menopause-Specific Quality of Life (MENQOL) questionnaire was used to collect the data, which refers to menopausal symptoms and how they affect the women's quality of life.

Results

Among menopausal symptoms, feeling anxious or nervous (64.7%) and changes in skin appearance, texture, or tone (8%) were the most and least common symptoms, respectively. Women taking medication for various health problems had higher levels of psychosocial (p = 0.005) and physical symptoms (p = 0.003) and a higher overall MENQOL score compared to women not taking medication. The results of the present study concerning the correlation of the vasomotor, psychosocial, physical, and sexual dimensions, both with each other and with the overall MENQOL score, were statistically significant. This means that an increase in one score causes the rest to increase, thus affecting the quality of life of menopausal women overall.

Conclusions

Menopause is associated with multiple symptoms that affect women's quality of life. Health professionals should inform menopausal women about methods and treatments that will help them have a better quality of life. More studies should be conducted to investigate the role of demographic and social factors that affect how women perceive the severity, intensity, and resolution of menopausal symptoms.

## Introduction

The irreversible termination of ovarian function and the lack of menstruation for at least 12 months are the hallmarks of menopause, according to the World Health Organization [1-2]. Menopause is a normal change in a woman's life [3], and the average age of onset is 51 years [4-5]. Menopause is a normal condition in a woman's life that is accompanied by vascular, physical, and psychological disorders, affecting her quality of life and affecting her health [6-9]. One of the most commonly reported symptoms by menopausal women is vasomotor symptoms [9]. The main vascular symptoms are hot flashes and night sweats. Also, the woman's mood during the transition to menopause is strongly linked to her quality of life during this period. Compared to pre-menopausal women, menopausal women experience a wide range of psychological symptoms, including mood disorders such as irritability, anxiety, sadness, and depression, which reduce women's quality of life [10].

Various social and demographic factors affect the intensity and frequency of menopausal symptoms as well as women's quality of life in general. The quality of life of each person is characterized by objective criteria (indicators of physical health and functionality) as well as by subjective criteria (psychological and environmental parameters) [11].

Factors such as the woman's marital status, economic level, educational level, physical activity, temperament, and the life attitude with which she chooses to manage menopause greatly influence the intensity and frequency of the menopause symptoms experienced and her level of quality of life [12]. Various studies have shown that a set of factors affects the quality of life of menopausal women [8,13].

Aim

This study aims to identify and quantify the most common symptoms experienced by menopausal women and to assess the impact of these symptoms on the overall quality of life of menopausal women living in a rural area of Greece.

The objective of this study is to give the opportunity to menopause women who live in small rural areas in Greece to participate in a scientific study to investigate the relationship between the severity of menopausal symptoms and factors such as age, ethnicity, socio-economic status, lifestyle, and access to health care in rural areas and to contribute meaningfully to the development of recommendations for improvement access to health care and support for these women.

## Materials and methods

The randomized sample of the present study consists of 150 women who visited the public outpatient gynecological clinics of the Aliveri State Health Center, Evia, a rural region. The participants are Greek women aged 40-60 years. They were informed about the purpose of the study, and they signed a consent/information form. The researchers visited the public outpatient gynecological clinics of the Aliveri State Health Center, Evia, every day from Monday to Friday and gave the questionnaire to every menopausal woman who was willing to participate. The collection of this data was performed in the second semester of 2022.

The inclusion criteria were as follows: a) menopausal women, b) agreement to participate in this study, and c) speak, understand, and read the Greek language.

The exclusion criteria were as follows: a) incomplete survey responses (participants who did not complete the entire questionnaire) and b) participants who refused to provide informed consent.

The data was collected using the anonymous Menopause-Specific Quality of Life (MENQOL) questionnaire, validated by Papadima et al., 2020, after the authors’ permission and includes 29 questions. Also, the questionnaire contained questions regarding the demographics of the women in the sample. Questionnaire responses are completed on a Likert scale from zero, not bothered at all, to six, extremely bothered [14].

The research was carried out following the granting of permission by the Fifth Ministry of Health of Thessaly and Central Greece, where the Aliveri State Health Center, Evia, is affiliated (No. Prot. 42819/18-5-2020).

Statistical analysis

We checked the normality of the quantitative variable distributions using the Kolmogorov-Smirnov test. To characterize qualitative variables, absolute (N) and relative (%) frequencies were employed. Two groups' quantitative variables were compared using the Student's test. The parametric test of analysis of variance (ANOVA) was utilized to compare quantitative variables between more than two groups. To examine the link between two quantitative variables, Pearson's correlation coefficient was employed. The two-sided significance threshold was established at 0.05 for statistical significance. The analysis was conducted using SPSS (IBM SPSS Statistics for Windows, IBM Corp., Version 22, Armonk, NY), a statistical tool.

## Results

The sample consisted of 150 women with a mean age of 52.3 years (SD = 2.3 years). The table below shows the demographics of the participants (Table 1).

**Table 1:** Demographic data of the women in the sample

		N (150)	Percentage (%)
Age		52.3 (2.3)	
Marital status	Married	125	83.3
	Divorced	13	8.7
	Widow	12	8.0
Children	Yes	146	97.3
	No	4	2.7
Number of children		2.2 (0.86)	2.0 (2.0-3.0)
Last menstrual period		50.7 (2.7)	51 (50-52)
Education status	Primary school	27	18.0
	Middle school	30	20.0
	High school	59	39.3
	Technological education	16	10.7
	University education	18	12.0
Professional status	Public officer	2	1.3
	Educational	7	4.7
	Freelancer	22	14.7
	Private officer	40	26.7
	Householder	65	43.3
	Retired	14	9.3
Sports	Yes	82	58.0
	No	63	42.0
Smoking	Yes	62	41.3
	No	88	58.7

One hundred twenty-five (83.3%) of the participating women were married, 146 (97.3%) had at least one child, and the median last menstrual period was 51 years (interquartile range (50.0-52.0)). In addition, the majority of 59 (39.3%) were high school graduates, and 65 (43.3%) were engaged in domestic work. Regarding health habits, 82 (58%) said they exercise, and 88 (58.7%) said they do not smoke.

The table below shows the scores for the vasomotor, psychosocial, physical, and sexual dimensions of the health and quality of life assessment of the participants. Higher values indicate more bothersome symptoms (Table 2).

**Table 2:** Vasomotor, psychosocial, physical, and sexual dimension symptoms

	Existence of symptoms	Intensity of symptoms		
	N (%)	Mean value (SD)	Median (interquartile range)	Cronbach's α
Vasomotor dimension	2.8 (2.4)		4 (0-5)	0.97
Hot flushes or flashes	95 (63.3)	3.1 (2.5)	0 (0-5)	
Night sweats	85 (56.7)	2.7 (2.5)	0 (0-5)	
Sweating	84 (56.0)	2.7 (2.5)	0 (0-5)	
Psychosocial dimension	2.2 (1.7)		2.1 (0.1-3.4)	0.81
Being dissatisfied with my personal life	90 (60.0)	3 (2.5)	0 (0-5)	
Feeling anxious or nervous	97 (64.7)	3 (2.4)	0 (0-5)	
Feeling depressed, down, or blue	45 (30.0)	2.6 (2.7)	0 (0-5)	
The feeling of waiting to be alone	64 (42.7)	2 (2.4)	0 (0-5)	
Accomplishing less than I used to	75 (50.0)	2 (2.5)	0 (0-5)	
Being inpatient with other people	58 (38.7)	1.6 (2.3)	0 (0-3)	
Experiencing poor memory	66 (44.0)	1.4 (2.2)	0 (0-4)	
Physical dimension	1.3 (1.1)		1.3 (01.3-2.1)	0.83
Feeling tired or worn out	12 (8.0)	2.5 (2.5)	0 (0-5)	
Difficulty sleeping	60 (40.0)	2.3 (2.6)	0 (0-5)	
Feeling a lack of energy	76 (50.7)	2.1 (2.4)	0 (0-4)	
Decrease in physical strength	68 (45.3)	2.1 (2.4)	0 (0-4)	
Decrease in stamina	44 (29.3)	2 (2.4)	0 (0-4)	
Aching in muscles and joints	69 (46.0)	1.8 (2.3)	0 (0-4)	
Low backache	69 (46.0)	1.5 (2.1)	0 (0-4)	
Aches in back of neck or head	72 (48.0)	1.3 (2.1)	0 (0-3)	
Weight gain	31 (20.7)	1.1 (2)	0 (0-3)	
Involuntary urination when laughing or coughing	40 (26.7)	1.1 (1.9)	0 (0-3)	
Dry skin	14 (9.3)	0.9 (1.8)	0 (0-0)	
Frequent urination	30 (20.0)	0.8 (1.7)	0 (0-0)	
Changes in the appearance texture or tone of your skin	12 (8.0)	0.7 (1.6)	0 (0-0)	
Increased facial hair	52 (34.7)	0.4 (1.2)	0 (0-0)	
Feeling bloated	29 (19.3)	0.4 (1.2)	0 (0-0)	
Flatulence (wind) or gas pains	40 (26.7)	0.3 (1.2)	0 (0-0)	
Sexual dimension		2.3 (1.9)	2 (2-4)	0.7
Vaginal dryness during intercourse	73 (48.7)	2.7 (2.5)	0 (0-5)	
Change in your sexual desire	82 (54.7)	2.1 (2.4)	0 (0-4)	
Avoiding intimacy	73 (48.7)	2.1 (2.4)	0 (0-4)	

**Figure 1:**
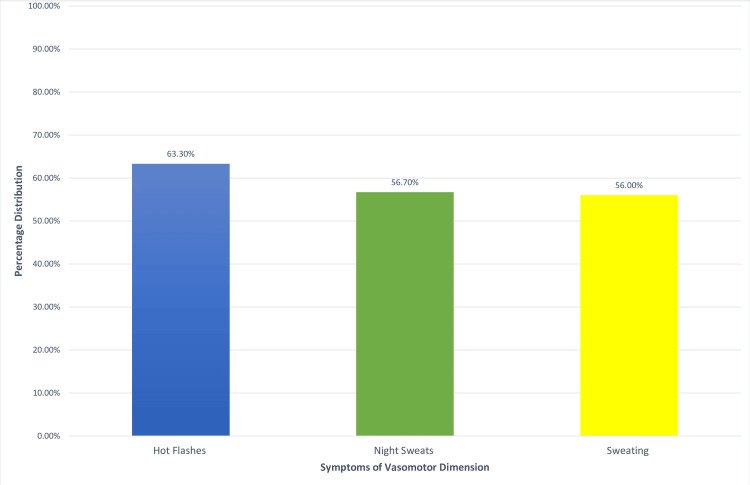
Percentage of women with symptoms from vasomotor dimension

The score on the vasomotor dimension scale had a mean value of 2.8 points (SD = 2.4 points). More frequent was the presence of symptoms related to hot flashes (95, 63.3%), and less frequent was the presence of night sweats (85, 56.7%) and sweating in general (84, 56.0%). The symptoms with the highest intensity (highest mean) were also those of hot flashes (mean = 3.1 points, SD = 2.5) (Figure 1).

**Figure 2:**
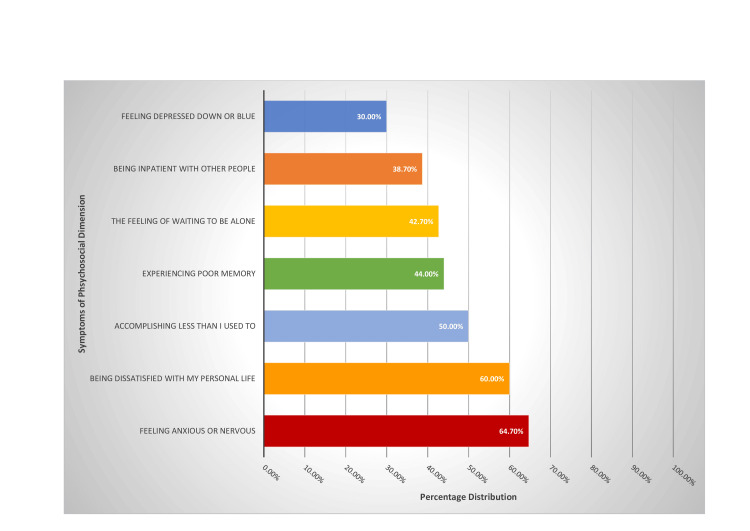
Percentage of women with symptoms from the psychosocial dimension

The score on the scale of the psychosocial dimension had a mean value of 2.2 points (SD = 1.7 points). The most frequent symptoms that women indicated were as follows: feeling anxious or nervous (97, 64.7%), I am dissatisfied with my personal life (90, 60%), and achieving less than I used to (75, 50%). The less frequent symptoms that women indicated were feeling depressed, down, or blue (45, 30%) and I am impatient with other people (58, 38.7%). The symptoms that had the highest intensity (highest mean) were as follows: I am dissatisfied with my personal life (mean = 3.0 points, SD = 2.5) and feeling anxious or nervous (mean = 3.0 points, SD = 2.4) (Figure 2).

**Figure 3:**
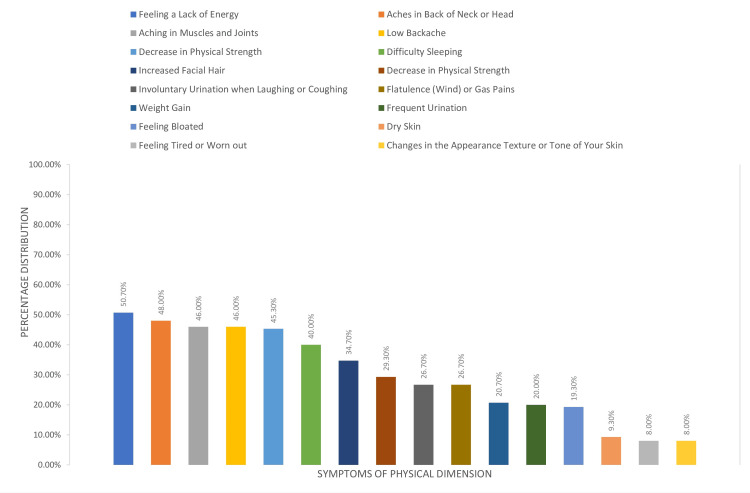
Percentage of women with symptoms from the physical dimension

The score on the physical dimension scale had a mean value of 1.3 points (SD = 1.1 points). More frequent was the presence of symptoms: feeling of lack of energy (76, 50.7%), pains in the neck or head (72, 48%), pains in the joints (69, 46%), or in the back (69, 46%), and less frequent was the presence of symptoms: feeling tired or exhausted and changes in skin appearance or texture (12, 8.0%). The symptoms that had the highest intensity (highest mean) were as follows: feeling tired or exhausted (mean value = 2.5 points, SD = 2.5) and difficulty sleeping (mean value = 2.3 points, SD = 2.6) (Figure 3).

**Figure 4:**
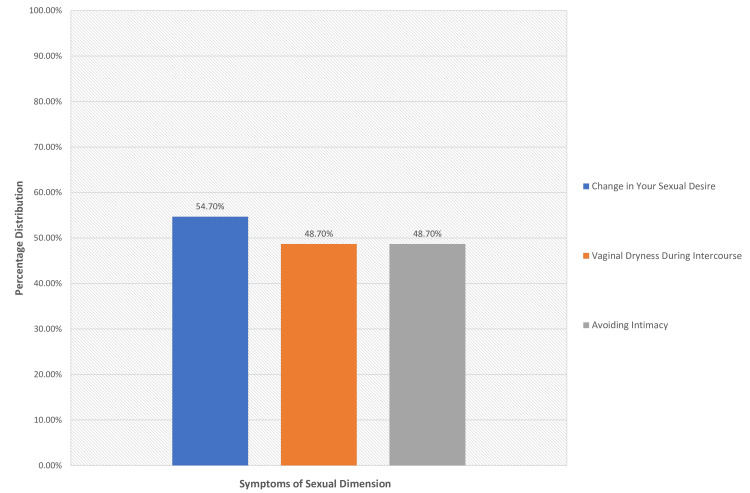
Percentage of women with symptoms of sexual dimension

The score on the sexual dimension scale had a mean value of 2.3 points (SD = 1.9 points). More frequent was the presence of the symptoms: change in sexual desire (82, 54.7%). Less frequent was the presence of the symptoms: avoidance of intimacy/sexual relationship (73, 48.7%) and vaginal dryness during intercourse (73, 48.7%). The symptom that had the highest intensity (highest mean) was vaginal dryness during intercourse (mean = 2.7 points, SD = 2.5) (Figure 4).

For all dimensions, Cronbach's α reliability coefficient was at least 0.7, indicating acceptable reliability.

A multivariate linear regression was then performed, with the vasomotor, psychosocial, physical, and sexual dimensions as the dependent variables and the demographic data of the participants as the independent variables.

Only the work factor was found to be significantly related to the vasomotor dimension. Specifically, working participants reported 0.89 points (p = 0.023) more discomfort than non-working participants (Table 3).

**Table 3:** Multivariate linear regression of the vasomotor dimension as dependent variable and the demographic data of the women as independent variable +dependence coefficient, ++standard error

Working woman	β+	SE++	p-value
No (report)	0.89	0.39	0.023
Yes	-	-	-

Smoking and medication factors were found to be independently associated with the psychosocial dimension. Specifically, women who took medication scored an average of 0.77 points (p = 0.005) higher than those who did not take it. Thus, taking medication is associated with more intense psychosocial symptoms.

Those women who smoked had an average of 0.68 points (p = 0.014) more reduced score compared to those who did not smoke. Thus, smoking is associated with milder psychosocial symptoms (Table 4).

**Table 4:** Multivariate linear regression with the psychosocial dimension as dependent variable and women's demographics as independent variables +dependence coefficient, ++standard error

	β+	SE++	p-value
Medicines			
No (report)	0.77	0.27	0.005
Yes			
Smoking			
No (report)	-0.68	0.27	0.014
Yes			

Also, the medication intake factor was found to be significantly related to the physical dimension. Specifically, participants who received medication scored an average of 0.52 points (p = 0.003) higher than those who did not receive it. Thus, taking medication is associated with more intense physical symptoms (Table 5).

**Table 5:** Multivariate linear regression with physical dimension as dependent variable and women's demographics as independent variables +dependence coefficient, ++standard error

Medicines	β+	SE++	p-value
No (report)	0.52	0.17	0.003
Yes	-	-	-

In addition, the age of the last menstrual period was found to be significantly related to the sexual dimension. Specifically, older participants had a lower score on this dimension. Thus, older age at the last menstrual period is associated with milder symptoms (p = 0.028) (Table 6).

**Table 6:** Multivariate linear regression with the sexual dimension as the dependent variable and the women's demographics as independent variables +dependence coefficient, ++standard error

	β+	SE++	p-value
Age of last menstrual period	-0.12	0.06	0.028

Below is the table of participants' scores on the MENQOL total scale according to their demographics (Table 7).

**Table 7:** Correlation of total MENQOL with women's demographics +Student’s t-test, ++ANOVA MANQOL, Menopause-Specific Quality of Life

		MENQOL total score		
		Mean value	SD	p-value
Married	No	1.7	1.1	0.713+
	Yes	1.8	1.2	
Children	No	1.5	1.8	0.583+
	Yes	1.8	1.1	
Education status	Primary/middle school	1.9	1.2	0.640++
	High school	1.7	1.2	
	Technological/university education	1.7	1.0	
Working woman	No	1.8	1.1	0.542+
	Yes	1.9	1.2	
Sports	No	1.8	1.1	0.800+
	Yes	1.8	1.2	
Smoking	No	1.8	1.1	0.681+
	Yes	1.8	1.2	
Medicines	No	1.6	1.1	0.002+
	Yes	2.1	1.2	

The total MENQOL score was found to be statistically significantly related to whether or not medication was taken. Specifically, women who take medication score higher, on average, than those who do not. Thus, taking medication is generally associated with more severe symptoms.

Multivariate linear regression was then performed with the MENQOL total score as the dependent variable and participant demographics as the independent variables. With the stepwise method, the results of the table below were found (Table 8).

**Table 8:** Multivariate linear regression with MENQOL total score as dependent variable and women's demographics as independent variables +dependence coefficient ++standard error MANQOL, Menopause-Specific Quality of Life

Medicines	β+	SE++	p-value
No (report)	0.58	0.19	0.002
Yes	-	-	-

Only the factor of medication intake was found to be significantly related to the total MENQOL score. Specifically, participants who received medication scored an average of 0.58 points (p = 0.002) higher than those who did not. Therefore, taking medication is generally associated with more severe symptoms.

Correlation of MENQOL dimensions and total score

The following table shows the correlations of the "vasomotor," "psychosocial," "physical," and "sexual" dimensions, both with each other and with the total MENQOL score (Table 9).

**Table 9:** Correlation of the vasomotor, psychosocial, physical, and sexual dimensions with each other and with the total MENQOL score +Pearson's correlation coefficient, P: p-value

		Vasomotor dimension	Psychosocial dimension	Physical dimension	Sexual dimension	Total score
Vasomotor dimension	r+	1	0.32	0.33	0.22	0.53
	P	-	<0.001	<0.001	0.006	<0.001
Psychosocial dimension	r+	-	1	0.66	0.55	0.85
	P	-	-	<0.001	<0.001	<0.001
Physical dimension	r+	-	-	1	0.56	0.91
	P	-	-	-	<0.001	<0.001
Sexual dimension	r+	-	-	-	1	0.70
	P	-	-	-	-	<0.001

All correlations were found to be statistically significant. Also, they are all positive, so increasing one score causes the rest to increase. This means that a woman who reports high symptom bother in one dimension is expected to report increased bother in the others as well.

**Figure 5:**
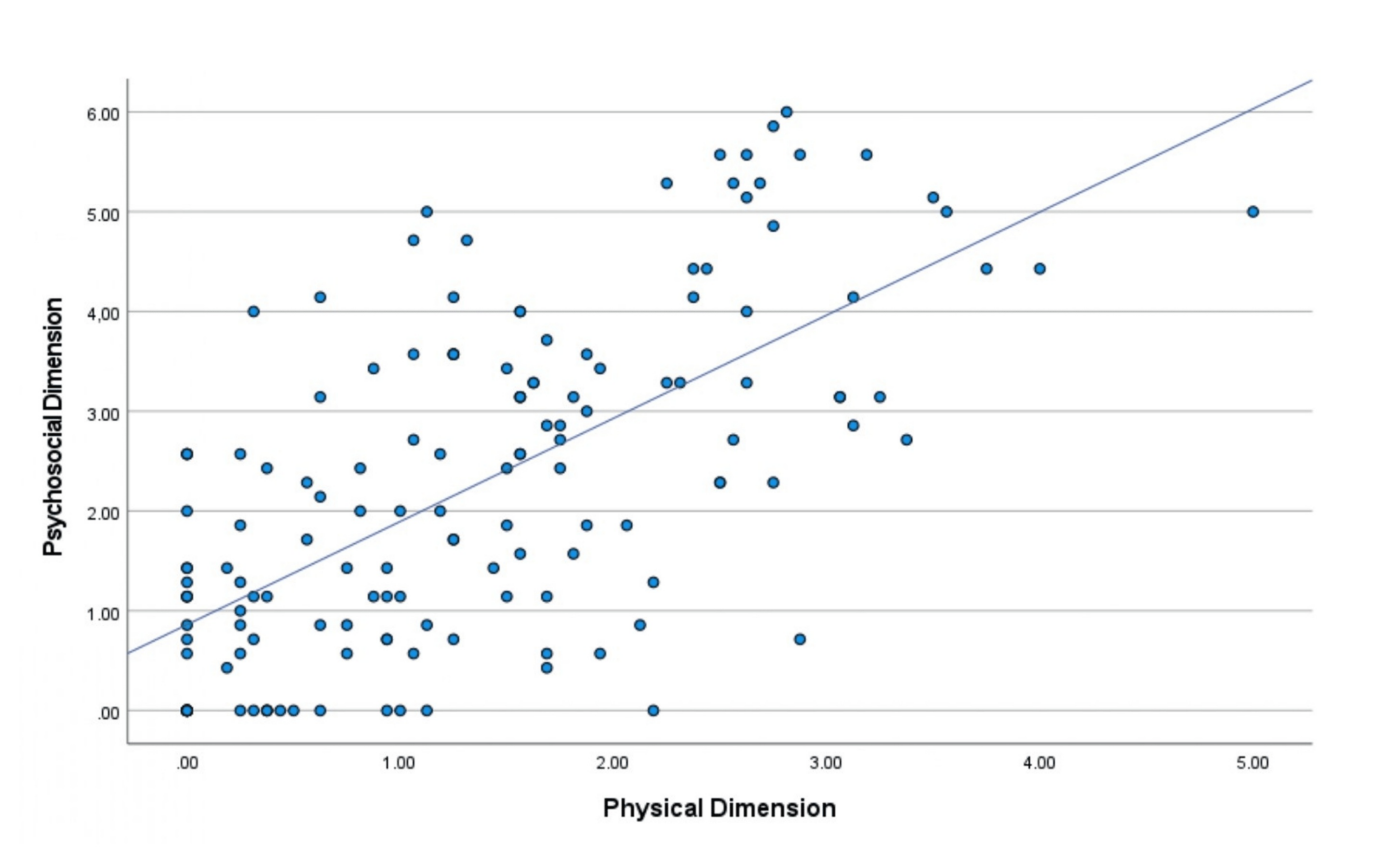
Diagrammatic illustration of the correlation between the psychosocial dimension and the physical dimension

**Figure 6:**
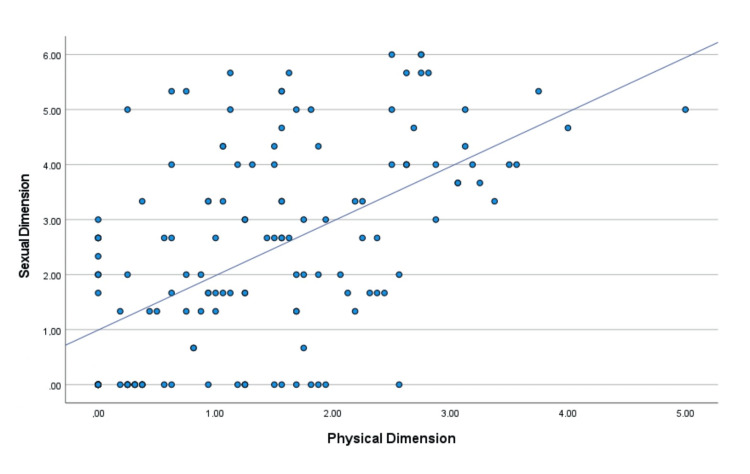
Diagrammatic illustration of the correlation between the sexual dimension and the physical dimension

**Figure 7:**
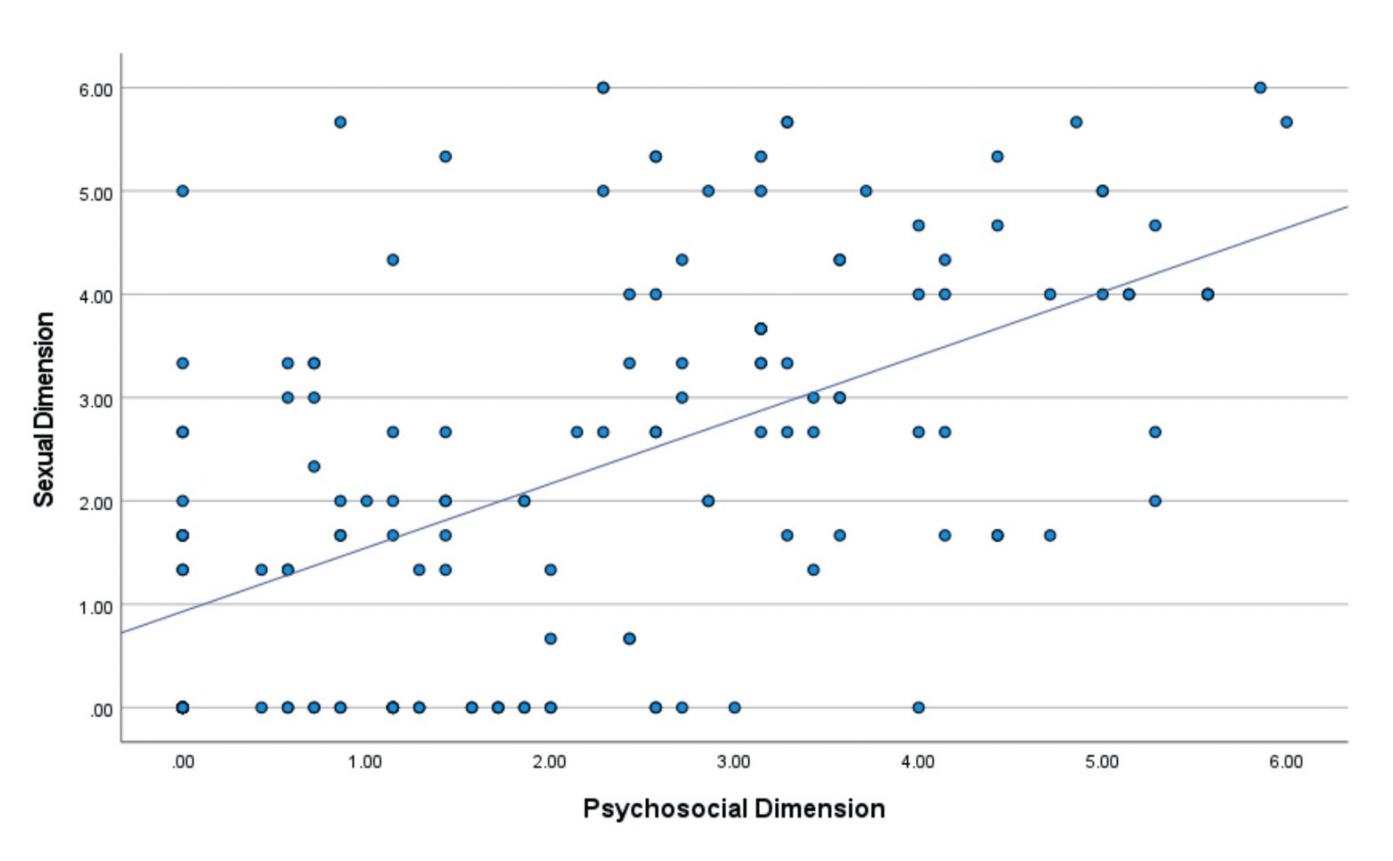
Diagrammatic illustration of the correlation between the sexual dimension and the psychosocial dimension

Specifically, the correlations were higher between the "psychosocial" and "physical" dimensions (Figure 5), the "sexual" and "physical" dimensions (Figure 6), and the "sexual" and "psychosocial" dimensions (Figure 7).

## Discussion

The present study investigated the severity of menopausal symptoms and their effect on the quality of life of women in a rural area of Greece. Also, the relationship of vascular, physical, psychosocial, and sexual symptoms with age, social, educational, family, and professional level, as well as with other factors such as exercise, smoking, and medication, was investigated. Finding and being able to approach menopausal women for sample collection in a small rural area of Greece is difficult. On the one hand, the population of most rural areas is small and quite elderly, and access to health centers is not easy. The health centers are located at a great distance from the villages, making it difficult for menopausal women to come to them. In addition, women, knowing that they are going through the transitional period of menopause due to their age, did not consider it necessary to visit the doctor and did not wish to report problems such as sexual disorders, sleep disorders, and mood swings. To collect the data, the validated MENQOL questionnaire was used, which makes it possible to identify and quantify symptoms of the physical dimension, the vasomotor dimension, the psychosocial dimension, and the sexual dimension. The results of the present study showed that the largest percentage of menopausal women from rural areas who visited the Aliveri State Health Center, Evia were married, and 83.3% and 97.3% of them had given birth to two to three children, respectively. Also, 39.3% had finished high school, and 43.3% were engaged in housework. The above results of the social and demographic data of menopausal women follow the traditional pattern of Greek women who live in rural suburbans.

The mean age in the last period was 50.7 years, and the most frequent vasomotor symptoms reported by participants were hot flashes, 63.3%. Also, high values were found in the psychosocial dimension with “feeling of anxiety or nervous” 64.7% and “being dissatisfied with my personal life” 60% while the other values of the psychosocial dimension are at high levels, “accomplishing less than I used” (50%) and “the feeling of waiting to be alone” (42.7%). The sexual dimension follows with high values of “change in your sexual desire” (54.7%), “vaginal dryness during intercourse” (48.7%), and “avoiding intimacy” (48.7%). On the contrary, mild symptoms were reported in the physical dimension, with the most frequent symptoms being “aching in muscles and joints” 48% and “low backache” 46%. Thus, we notice that despite the intense symptoms faced by the menopausal women in the countryside in their sexual life as well as in their daily lives, from hot flashes, muscle pains, and psycho-emotional changes, they did not seek medical help to improve or solve the problems. The same observation was made in a study by Blümel et al., where they reported that menopausal women are more likely to experience vasomotor (10.6 times), psychosocial (3.5 times), physical/physical (5.7 times), and sexual (3.2 times) disorders than the rest women [15], leading to a lower quality of life for these women [12].

The relationship between work and the quality of life of menopausal women is the subject of study by many researchers. Research shows that working women report a better quality of life than those who do not work [13]. In the present study, correlating independent variables with the symptoms of each dimension separately, the results showed that the working participants women presented high levels of discomfort from the symptoms of the vasomotor dimension compared to the other participants. Working women in rural areas are engaged in agricultural and livestock work and then during the day with household duties. The result of a hard daily life has more intense vasomotor symptoms but did not differentiate the total score of the quality of life of working women compared to non-working women. Alizadeh-Charandabi et al., in their research, reported that working women scored higher than those who were not working but without a significant statistical difference [16]. In the same study, they reported that although working women had a better quality of life, work alone could not be a "predictor" of quality of life, nor the only effective factor in its improvement. Yoeli et al., in their study, found that women who worked in a high-level environment reported more vasomotor disorders as the main symptom that bothered them, while women with a casual work relationship reported musculoskeletal pain as the main symptom [17]. On the contrary, Shobeiri et al., in their research, showed that no significant statistical correlation was found between working women and those who were involved in household chores [18]. It is unknown exactly how a woman's employment influences how severe her vasomotor symptoms are. It is possible that food and lifestyle choices, along with social and psychological aspects, have a significant impact on this outcome [4].

Studies have shown that there is a significant relationship between marital status and a woman's quality of life, as well as between marital status and the frequency and severity of menopausal symptoms [19]. Married women report a better level of quality of life compared to that of singles and widows [20]. In their study, Ehsanpour et al. found that there is a correlation between the psychological dimension and the woman's marital status, as well as between the sexual dimension and her marital status [21]. In contrast, no correlation was found between the woman's marital status and the social and physical dimensions. In the present study, it was found that the woman's marital status had no significant statistical correlation with any of the four dimensions of quality of life. The present finding would have the possibility to be used and explored in future research and to look for the reasons that led to this result, such as if they themselves communicated their concerns or physical problems and sought help from family, friends, and experts, respectively. In addition, the result of the present study may be due to the small number of married women who participated in the research. Research conducted on 236 postmenopausal women in Iran in 2022 showed a similar result [20].

According to Norozi et al., a woman's level of education is an important factor that positively influences the severity of menopausal symptoms. In the present study, no significant statistical correlation was found between the level of education and the woman's quality of life, which may be attributed to the small number of women with post-high school education [12]. Also, we should consider that the greater percentage of women living in rural areas of Greece receive a basic education. University-educated women leave small destinations due to a lack of jobs in scientific fields or large corporate financial centers. However, the research of Kafaei Atrian et al. had a similar effect in Iran [20].

The anti-estrogenic properties of smoking and nicotine dependence negatively affect the severity of menopausal symptoms [22]. Dotlic et al. found that there is insufficient evidence to link the number of cigarettes smoked per day and the duration of smoking with quality of life in postmenopausal women [23]. In the same study, which included 513 postmenopausal smokers, occasional smokers, and non-smokers from Belgrade, it was found that longer duration of smoking and not the number of cigarettes smoked per day had a negative effect on the physical dimension and the total MENQOL score. In the present study, female smokers reported milder symptoms in the psychosocial dimension compared to non-smoking females. This finding needs further investigation to ascertain the causes and factors. They were not asked about the number of cigarettes smoked per day and the duration of smoking. One possible explanation is that these women smoked occasionally when or whenever they felt stressed or anxious.

The present study involved women who were taking medication for health problems such as hypothyroidism, diabetes, hypertension, arrhythmias, and depression. These women had a higher score in the psychosocial and physical dimensions, as well as in the total MENQOL score. That is, they reported that they had more intense psychosocial symptoms by 0.77 points, more intense physical symptoms by 0.52 points, and in general, they experienced more intense symptoms by 0.58 points compared to the symptoms reported by women who did not receive treatment. Thornton et al., in their research, found that women taking medication for various health problems, such as diabetes, depression, and heart problems, reported higher scores on the sexual dimension [24]. The data demonstrate the effect that drugs have on women's quality of life as they alter their psychosocial and physical state.

Age at last menstrual period was found to be significantly associated with sexual orientation in the present study. Women with older menarche had milder sexual symptoms. Milder symptoms and better quality of life were also found in the research by Kafaei Atrian et al. in 2018 [20].

The correlations of all four dimensions, both with each other and with the overall MENQOL, emerged as statistically significant. This means that a woman who declares great discomfort from symptoms in one dimension is expected to declare increased discomfort in the remaining dimensions as well, with the result that her quality of life is negatively affected. The effect of menopausal symptoms acts like a domino effect. The strong impact of menopausal symptoms on the quality of life of menopausal women is proven worldwide. Women need an individualized approach depending on the intensity and frequency of symptoms depending on the causes and age, beliefs, and specificities. Continuous information and sampling campaigns will enable targeted improvement in supporting women and solving their problems.

The novelty of the present study is the collection of a sample of menopausal women from remote villages of the Aliveri district. The participating women were taking part in a scientific study for the first time. Also, it was the first time that they reported personal data about their psychosocial and sexual life. The findings may not be generalizable to women with different socioeconomic and cultural backgrounds due to the small number of participants. In this study, the randomized sample was only women of Greek nationality, so we did not have the opportunity to study valuable insights from women of different nationalities.

## Conclusions

Menopause is a complex phenomenon in a woman's life. It is affected by a multitude of factors and is experienced differently by each woman. Our study showed that women taking medication for various health problems had higher levels of psychosocial and physical symptoms. Also, the results of our study showed that an important factor of milder symptoms in the sexual dimension was the year of the last menstrual period; the older the woman was, the milder the symptoms were. The results of the present study regarding the association of the vasomotor, psychosocial, physical, and sexual dimensions, both among themselves and with the total MENQOL score, were statistically significant. This means that an increase in one score causes the others to increase, thus affecting the quality of life of menopausal women as a whole.

The results of our study were important, underlining that women need gynecological care and support during menopause to prevent or even improve physical, psychosocial, vascular, and sexual disorders. Health professionals should inform and counsel women individually about menopause, considering health history, temperament, lifestyle, and reported symptoms.
